# Exploring the potential of Raman micro‐spectroscopy of radiochromic films for experimental microdosimetry

**DOI:** 10.1002/mp.17900

**Published:** 2025-07-15

**Authors:** Connor McNairn, Prarthana Pasricha, Kirsty Milligan, Iymad R. Mansour, Edana Cassol, Vinita Chauhan, Jeffrey L. Andrews, Sanjeena Subedi, Andrew Jirasek, Bryan R. Muir, Rowan M. Thomson, Sangeeta Murugkar

**Affiliations:** ^1^ Department of Physics Carleton University Ottawa Ontario Canada; ^2^ Department of Physics University of British Columbia ‐ Okanagan Campus Kelowna Canada; ^3^ Radiation Medicine Program Princess Margaret Cancer Centre Toronto Ontario Canada; ^4^ Department of Radiation Oncology University of Toronto Toronto Ontario Canada; ^5^ Department of Health Sciences Carleton University Ottawa Ontario Canada; ^6^ Consumer and Clinical Radiation Protection Bureau Health Canada Ottawa Ontario Canada; ^7^ Department of Statistics University of British Columbia University of British Columbia ‐ Okanagan Campus Kelowna Canada; ^8^ Department of Statistics Carleton University Ottawa Ontario Canada; ^9^ Metrology Research Centre National Research Council of Canada Ottawa Ontario Canada

**Keywords:** film heterogeneity, high spatial resolution dosimetry, radiochromic film, Raman micro‐spectroscopy

## Abstract

**Background:**

Micrometer‐scale dosimetry is crucial when estimating the energy deposited within micrometer‐scale biological targets exposed to low doses or high dose gradients. Raman micro‐spectroscopy read‐out of radiochromic films (RCFs) permits micrometer‐scale resolution; this presents a novel opportunity to explore its feasibility for experimental microdosimetry.

**Purpose:**

The purpose of this work was to develop a novel approach towards generating data for experimental microdosimetry. The objective was to develop a method based on high (1–2 µm) spatial resolution Raman micro‐spectroscopy of RCFs, ensuring reproducibility of data while producing two‐dimensional intensity maps of the Raman response.

**Methods:**

EBT3 RCFs were irradiated to doses between 0.2 Gy and 2 Gy using a clinical linear accelerator. Raman spectra were collected using a custom Raman microscope fitted with 40× and 60× water immersion (WI) objectives, and a commercial Raman microscope utilizing a 100× dry objective. The excitation source of the custom setup was circularly polarized to minimize the influence of polarization on the film read‐out. The Raman response of the RCFs was measured over a 100 × 100 µm^2^ region of interest (ROI) with a 10 × 10 grid. The Raman response of the active layer of the film was normalized to the radiation‐insensitive monomer peak at 2260 cm^−1^. The Raman intensities of the 1445 cm^−1^ and 2060 cm^−1^ peaks were used to generate dose response curves for each microscope setup. Maps of the Raman intensity over the 100 × 100 µm^2^ ROI for the 60× WI setup were used to quantify the heterogeneity in the film response. Higher resolution point‐scans were performed over a 20 × 20 µm^2^ ROI for 0 Gy and 2 Gy samples.

**Results:**

The dose response of each Raman microscope setup over the 0–2 Gy dose range was linear (*r* ^2^ of 0.98) after normalization to the 2260 cm^−1^ Raman peak in the active layer. The slope of the dose response curve of the commercial microscope exhibited dependence on the film orientation; this was minimized with the custom Raman setup by using the circularly polarized excitation source. The relative standard deviation (RSD) of the 1445 cm^−1^ peak Raman intensity over the 100 × 100 µm^2^ ROIs was significant (∼11%) for each microscope setup, and independent of dose. The Raman intensity distribution maps revealed that the heterogeneity in Raman response across the ROI was on the same size‐scale (1.62 µm × 9.4 µm) of the lithium salt of pentacosa‐10,12‐diynoic acid (LiPCDA) crystals comprising the active layer of the film.

**Conclusions:**

This work explored the feasibility of a Raman micro‐spectroscopy‐based read‐out technique of RCFs for experimental microdosimetry. Utilizing the 2260 cm^−1^ peak in the Raman spectrum as an internal standard for normalization produced a linear (*r* ^2^ of 0.98) dose‐response curve in the 0–2 Gy dose range. Utilizing circularly polarized laser excitation minimized the polarization dependence of the film and increased the reproducibility of the Raman measurements. Spatial heterogeneity in the concentration of PCDA crystals in the active layer was visualized based on two‐dimensional maps of the Raman intensity response to explore the implications on microdosimetry.

## 1 INTRODUCTION

Radiochromic films (RCFs) are widely used dosimeters that are promising for the development of a high‐spatial resolution, micrometer‐scale dosimetry technique due to their tissue equivalence and energy‐independent response. The sensitive layers of RCFs are comprised of monomer crystals of pentacosa‐10,12‐diynoic acid (PCDA) that are dispersed throughout the active layer of the film. Upon exposure to ionizing radiation, these crystals polymerize and form long‐chain polymers known as polyPCDA. The greater the quantity of polyPCDA polymers, the more light they absorb.^1^, ^2^ The absorbed dose delivered to these films is typically read out using flatbed optical scanners, which measure the change in optical density of the films. However, these optical scanners are limited to resolutions ranging between 85 and 350 µm, which is not sufficient for micrometer‐scale spatial resolution.^3^, ^4^

Mapping of doses with a spatial resolution on the order of micrometers is useful for diverse contexts across a broad dose range. Emerging radiotherapy techniques, such as microbeam radiation therapy, which deliver arrays of pencil beams on the order of 10s of microns in diameter, require micrometer‐scale resolution to accurately assess delivered dose involving large dose gradients.^5^, ^6^ Low doses of radiation (< 0.1 Gy) to healthy tissues are relevant to radiation protection applications, for example, in x‐ray imaging and radiation therapy. In particular, the links between low‐dose exposures and increased health risks are unclear, and there remains a need to understand the fundamental mechanisms of cellular response at low doses.^7^ At these low doses, and considering micrometer‐scale cellular volumes, microdosimetry needs to be considered. Microdosimetry provides the theoretical framework for considering energy deposition in these contexts, defining the specific energy as the energy imparted per unit mass within a volume of interest or target. Specific energy is the stochastic analog of the absorbed dose (which is generally equal to the mean specific energy). When considering a population of cellular targets at low doses, there will be considerable variation in specific energy imparted in these micrometer‐scale targets, and some targets may receive no energy.^8^ As a specific example, for mammography, the standard deviation of the specific energy for mammary epithelial cell nuclei is 85% relative to the mean.^9^ These considerations underscore the need for micrometer‐scale resolution to assess specific energy imparted for doses in the 0–2 Gy range.^10^ Moreover, a technique that allows the identification and separation of a small number of irradiated cells at low doses from the nontargeted bystander cells in the population is highly desirable.

Raman spectroscopy involves the inelastic scattering of light resulting from interactions with specific vibrational modes of chemical bonds in molecules. As such, Raman spectroscopy allows multiplexed chemical information to be collected simultaneously, capturing a “chemical fingerprint” corresponding to multiple macromolecules, for example, DNA, lipids, and proteins.^11^ Raman spectroscopy is a nondestructive and label‐free technique that has been utilized for developing cancer diagnostics.^12^ Raman micro‐spectroscopy has been applied to investigate the fundamental mechanisms of radiobiological response in cells and tissue^13^, ^14^, ^15^ and to detect both targeted and nontargeted effects of ionizing radiation damage in vitro.^16^ In addition, it has also been shown to be a promising readout method for RCFs to achieve both micrometer‐scale spatial resolution and sensitivity over a range of doses as high as 50 Gy.^1^, ^17^, ^18^, ^19^, ^20^ Our recent work demonstrated a readout technique for radiochromic (EBT3) film samples based on Raman micro‐spectroscopy with a spatial resolution of 30 µm and provided improved sensitivity to doses as low as 0.03 Gy, while investigating an extended range of doses up to 50 Gy for the dose‐response curves.^20^ Although microscopes and microdensitometers have been employed to evaluate the dose profile of pencil beams at a resolution as high as 1.5 µm and ∼20 µm, respectively,^5^, ^6^ those readout methods cannot assess radiation‐induced biochemical changes in cells without the use of one or more external contrast agents which involves additional sample processing steps. Thus, Raman micro‐spectroscopy offers the potential for the development of an experimental microdosimetry technique to not only detect radiobiological changes in cells but also to identify and estimate the dose delivered in targeted cells exposed to low doses as well as small field sizes based on the micrometer‐resolution Raman response of RCFs.

In this study, we develop a novel approach towards generating data for experimental microdosimetry, which requires measurement sensitivity, reproducibility, and evaluation of spatial heterogeneity at low doses. Similar to prior reports,^17^, ^18^, ^19^, ^20^ we use Raman micro‐spectroscopy based on a point‐scanning technique with 1–2 µm lateral spatial resolution to read out the Raman intensity response over a 100 × 100 µm^2^ area of EBT3 samples of RCF. However, in contrast to previous work involving micrometer‐resolution Raman spectroscopy of RCFs,^6^, ^17^, ^18^, ^19^, ^20^ our work demonstrates for the first time, (1) the use of circularly polarized excitation light to minimize the polarization dependence of the Raman response of RCF samples; (2) the use of the radiation‐insensitive 2260 cm^−1^ Raman peak in the active layer of the RCF as an internal standard for normalization of the Raman spectral intensity; and (3) generation of two‐dimensional maps of the Raman intensity of individual pixels to quantify the heterogeneity of the Raman response of the RCF samples. We test the robustness of our approach by utilizing three different volumes of the focused laser spot and using two different Raman instruments: a commercial Raman microscope and a custom Raman micro‐spectroscopy setup. We show that our method of using circularly polarized light for excitation and the internal standard for normalization of the Raman response improves the reproducibility of the Raman micro‐spectroscopy‐based readout of RCFs and facilitates inter‐ and intra‐microscope comparisons. Moreover, it provides a linear dose‐response resulting in improved dose resolution in RCFs irradiated at low‐moderate doses between 0.2 Gy and 2 Gy. We utilize the results of the spatial heterogeneity found from the two‐dimensional Raman intensity maps of RCFs to discuss the implications for experimental microdosimetry.

## 2 MATERIALS AND METHODS

### 2.1 RCFs

**FIGURE 1:**
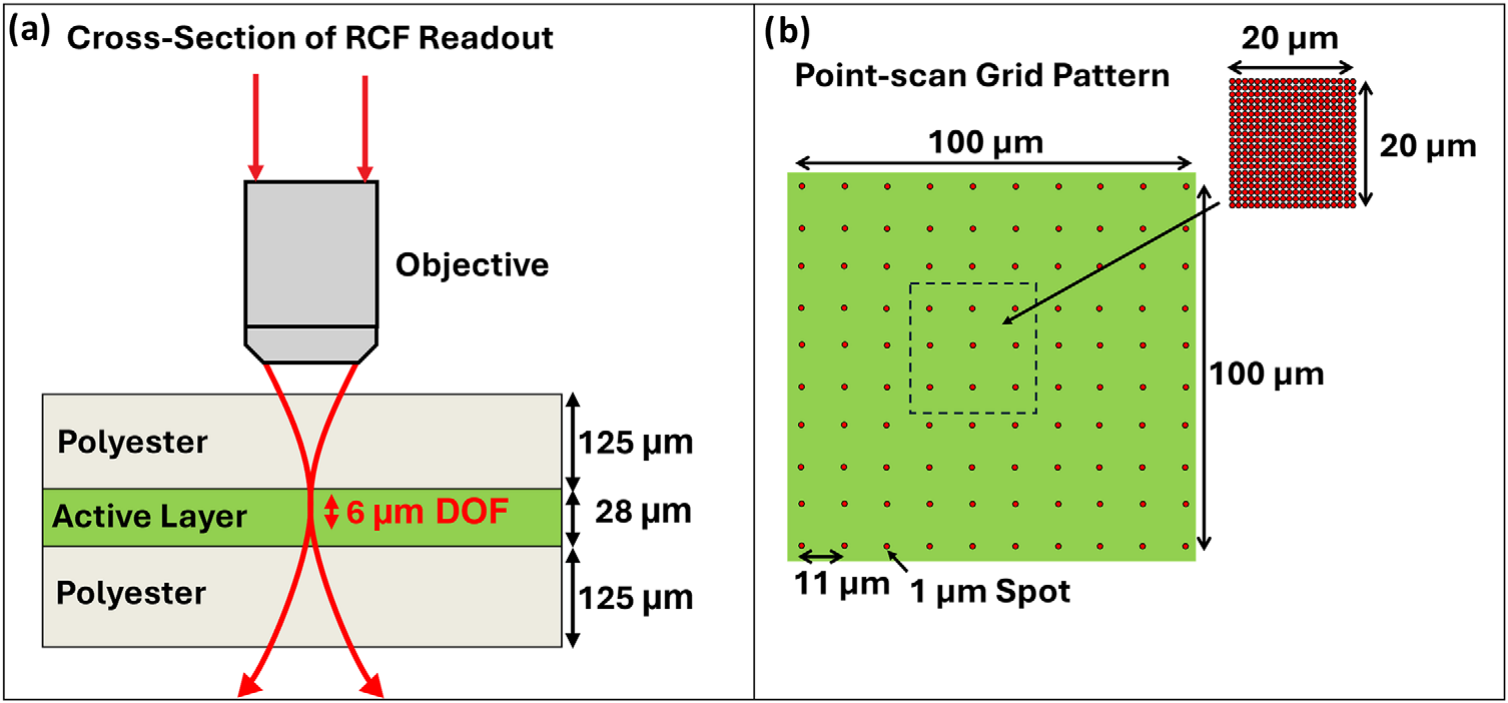
(a) Cross‐section of RCF compared to the depth of focus of the microscope. (b) Schematic of 10 × 10 grid scan over 100 × 100 µm^2^ ROI, each point has a spatial resolution of 1 µm and is separated from adjacent points by 11 µm. The inset shows the schematic of a 20 × 20 grid scan over a 20 × 20 µm^2^ ROI, where each point has a spatial resolution of 1 µm and is directly adjacent to nearby points. ROI, region of interest.

The RCFs used in this study were GAFChromic EBT3 RCF (Ashland Specialty Ingredients, Chatham, NJ, USA). EBT3 films consist of a 28 µm active layer laminated by two 125 µm transparent polyester layers (Figure 1a). The active layer consists of crystals of LiPCDA dispersed throughout the layer.^1^ The films were cut into square pieces of area 2.5 × 2.5 cm^2^ from a single sheet, and marked to ensure the orientation of each sample was consistent. The films were kept either in darkness or in low light conditions to minimize light‐induced polymerization of the film's active layer.

### 2.2 Radiation exposure

Irradiations of GAFChromic EBT3 film samples were performed using the Elekta Synergy clinical linear accelerator at the National Research Council of Canada (NRCC), with a 6 MV photon beam, directed perpendicular to the surface of the film. Each irradiation involved placing a 2.5 × 2.5 cm^2^ sample of RCF at a depth of 5 cm, precisely at the center of a 30 × 30 cm^2^ Virtual Water (Med‐Cal, Inc., Coral Gables, FL, USA) phantom. To ensure sufficient backscatter, a 5 cm slab of virtual water downstream was placed below the film. Different options were investigated to produce an extended source‐to‐surface distance (SSD), including the use of a horizontal beam geometry that would allow for the delivery of lower doses.  The results of this work use an extended SSD of 214.8 cm implemented with a vertical beam geometry by positioning the virtual water phantom close to the floor. The field size was adjusted accordingly (collimator setting was 5 × 5 cm^2^) to deliver an approximately 10 × 10 cm^2^ field at the extended phantom surface for all doses. Samples of RCF were irradiated to doses of 0.2, 0.3, 0.4, 0.5, 0.6, 0.7, 0.8, 0.9, 1.0, 1.2, 1.4, 1.6, 1.8, 2.0 Gy, with a duplicate 1.0 Gy sample also created and two unirradiated (0 Gy) samples used as controls. The absorbed dose per monitor unit (MU) was measured using a calibrated secondary standard reference chamber (type PTW30013) connected to an electrometer (Keithley 6517A). To determine drift in linac output over the course of film irradiations, absorbed dose was measured using the reference chamber both before and after irradiations. The uncertainty in determination of doses greater than 0.5 Gy using this linac geometry at NRC is 0.6%, increasing to ∼2.6% for doses less than 0.5 Gy.^20^

### 2.3 Raman micro‐spectroscopy and readout of RCFs

Raman measurements of RCFs were performed using two different instruments for read‐out in order to determine the dependence of the RCF Raman response on the type of Raman instrumentation employed.

#### 2.3.1 Custom Raman microscope

The custom‐built Raman microscope is an evolution of the design used in our previous study.^20^ In particular, the 500 mW, 785 nm multimode laser was replaced with a more stable (<1% power fluctuation) single‐mode fiber‐coupled laser at 785 nm (Innovative Photonic Solutions, Plainsboro Township, NJ, USA). A laser diode controller (Thorlabs CLD1015) was used to produce a power output of ∼60 mW out of the laser. To remove any polarization dependence from the microscope, a quarter‐waveplate (Thorlabs, Newton, NJ, USA) was added in the collimated beam path to circularly polarize the light. To minimize laser‐induced polymerization, an optical neutral density filter was added to limit the average laser power to ∼4 mW at the RCF sample. Two different water‐immersion (WI) microscope objectives (Olympus, Richmond Hill, ON, Canada), a 60× (1.1 NA) and a 40× (0.8 NA) WI microscope objective, were used to collect data. This resulted in a focused single‐mode laser spot with a lateral diameter of ∼ 1 µm and depth of focus of ∼6 µm for the 60× WI objective (see Figure 1a) and a lateral diameter of ∼ 1.2 µm and depth of focus of ∼14 µm for the 40× WI objective. A point scan technique was achieved by automating the movement of the RCF sample through the focal volume of the microscope using a three‐axis automated stage (FTP 2000, ASI Inc., McLean, VA, USA) with a resolution of 22 nm. The back‐scattered Raman signal from the RCF sample passed through a dichroic mirror and two long‐pass edge filters (Iridian, Ottawa, ON, Canada) to reject the laser light. It was coupled into an optical fiber with a 100 µm diameter core which delivered the signal to a Raman spectrometer (Andor Shamrock 303i) using a 100 µm slit and a 600 line/mm grating. Raman spectra were measured in the 614–2313 cm^−1^ range, with a spectral resolution of ∼4 cm^−1^ using a CCD camera (Newton DU920P‐BEX2‐DD, Andor) that was thermoelectrically cooled to –80°C.

#### 2.3.2 Commercial Raman microscope

A commercial confocal Raman microscope (In Via, Renishaw Inc., Wotton‐under‐Edge, Gloucestershire, UK) set up was also used to allow for comparison with results obtained from the custom Raman microscope setup. This specific setup utilized a 100× dry objective (0.9 NA) (Leica Microsystems, Richmond Hill, ON, Canada), an 830 lines/mm diffraction grating, and a 1000–2300 cm^−1^ spectral window. Raman spectra were recorded with a thermoelectrically cooled CCD detector (Renishaw RenCam). A 785 nm single‐mode laser (Renishaw, Wotton‐under‐Edge, Gloucestershire, UK) was used for excitation. The laser power density at the sample was 0.5 mW/µm^3^ with a sampling volume of 2 × 5 lateral and 10 µm axial. A grid scan was achieved using a Renishaw encoded stage (model MS30), with 0.25 µm lateral resolution.

### 2.4 Raman read‐out operation based on point‐scanning technique

Methods used to collect Raman spectral data from the custom and commercial Raman microscopes were identical unless otherwise described. RCF samples were held in place on a custom‐made solid stainless‐steel slide to ensure flatness of the RCF samples and provide consistent film orientation. The position along the z‐axis was adjusted by first obtaining the focused back‐reflected laser spot from the RCF surface in the bright‐field camera image and then vertically translating the stage to maximize the Raman signal at 1445 cm^−1^ from the polymer in the active layer of the RCF. Raman spectra were obtained by automated X‐Y translation of the RCF sample through the laser focus using an automated stage. As illustrated in Figure 1b, data were sampled from a 10 × 10 grid (pixel) pattern with ∼11 µm separation between pixels over a 100 × 100 µm^2^ area, also referred to as the region of interest (ROI) in this work. Preliminary measurements of the unirradiated (control) RCF samples revealed a nonlinear intensity response as a function of the focused laser power at the RCF sample (data not shown). Previous studies have suggested that this may be a result of laser‐induced polymerization in the film.^1^ Hence, a neutral density filter was used to lower the laser power at the sample to avoid any artifacts in the Raman intensity response due to laser‐induced polymerization of the RCFs. An optimum laser excitation power was determined to be ∼4 mW, for which the active layer peak did not show an increase in the Raman intensity over the measurement timescale. This power is lower than what was used in our previous study^20^ due to the smaller focal volume employed here, requiring a lower power to avoid laser‐induced polymerization. A quarter‐wave plate was used to circularly polarize the laser and minimize any dependence of the Raman signal on the orientation of the sample with respect to the polarization of the laser.^17^ The data collection time per spectrum was set to 1 s (0.3 s for the commercial microscope), and at each pixel, five spectra were collected for a total of 500 spectra per sample. Raman measurements were performed in two ways: (i) all 0–2 Gy‐irradiated RCF samples were measured in a single day with the measurements of Raman datasets repeated in triplicate using both a 40× WI and 60× WI objective; and (ii) using a 100× dry objective (0.9 NA), 0–2 Gy‐irradiated RCF samples were collected over 5 days, with three replicates collected on the same day for each dose. A measurement of the Raman spectrum of a fluorescence standard (SRM 2241, National Institute of Standards and Technology, NIST, Gaithersburg, MA, USA)^21^ was taken twice within the testing period of the RCF samples. This was used to correct for the decreased sensitivity of the spectrometer at higher (> 1500 cm^−1^) Raman shifts. Standard samples of polystyrene and silicon were measured prior to each set of Raman measurements of RCFs.

To further investigate the spatial heterogeneity in the Raman response from RCFs, four measurements were performed using the custom Raman microscope on control and 2 Gy samples over a 20 × 20 µm^2^ ROI sampled using a 20 × 20 grid and the 60× WI objective. Unlike the previous readout grid (Figure 1b), each point (pixel) was directly adjacent to one another, as shown in the inset of Figure 1b. The measurements were taken at the corners of a 1 × 1 mm^2^ square area for both the control and 2 Gy samples to avoid overlap. Five spectra were collected with a 1 s collection time for each pixel for a total of 2000 spectra per ROI.

### 2.5 Raman spectral data preprocessing

Raman spectra were preprocessed using in‐house scripts based on MATLAB 2024b (MathWorks, Natick, MA, USA). A modified cosmic‐ray algorithm was used to remove artifacts caused by cosmic‐ray interactions.^22^ For each pixel, the five spectra were averaged to reduce the stochastic variation and obtain a representative spectrum for that pixel. A background subtraction using the sensitive nonlinear iterative peak‐clipping (SNIP) algorithm was applied to these data, followed by the NIST correction curve.^21^ The SNIP technique iteratively determines a baseline to subtract from the spectrum by identifying the minimum distance between a given point and the average value of the outer edges of a window centered on that point.^23^ For generating the dose response curves, Raman spectra at a given dose were first vector normalized to minimize variance in signal due to slight differences in measurement conditions for each RCF sample between datasets.

### 2.6 Normalization of film response to the 2260 cm^−1^ monomer peak in the active layer

The Raman intensity response of the 2260 cm^−1^ monomer peak in the active layer was determined to be constant at doses up to 30 Gy (see Section 3.1 and Figure S1). Prior reports suggest that significantly higher doses are required to fully polymerize diacetylenes and cause the 2260 cm^−1^ monomer peak to fully disappear from the Raman spectrum. For example, Melveger et al. found that 50 KGy resulted in 11.4% monomer to polymer conversion for a diacetylene similar to PCDA.^24^ Hence, to maximize differences between doses within a dataset, the Raman spectra were normalized to the intensity of this radiation‐insensitive Raman peak. As such, the 2260 cm^−1^ monomer peak served as an internal standard within the active layer of the film to normalize the film response. Normalization to the 2260 cm^−1^ will not completely eliminate the spatial heterogeneity of the polymer signal since the density of the polymer crystals and monomer crystals is not completely equivalent at every pixel in the ROI.

### 2.7 Comparison of Raman response of RCFs with linearly and circularly polarized laser excitation

A set of films irradiated with a representative set of doses (0, 0.5, 1.0, 1.6, 2.0 Gy) were read out using the point scanning technique with the custom Raman microscope to assess the impact of using circularly polarized versus linearly polarized excitation. The quarter‐wave plate was removed to use the linearly polarized laser, and each irradiated RCF sample was read out at mutually orthogonal (landscape and portrait) orientations. Using linearly polarized light, the landscape orientation of the samples corresponded to the higher Raman signal.^18^ To circularly polarize the source again, the quarter‐wave plate was reinstalled and the measurements repeated. Dose‐response curves were generated for each orientation and polarization.

### 2.8 Assessment of the heterogeneity of the Raman response to determine feasibility for experimental microdosimetry

Monte Carlo simulations^25^ can quantify the large variation in the specific energy imparted (microdosimetric spread) in micrometer‐scale targets at low doses of ionizing radiation. To quantify the corresponding spatial heterogeneity of the Raman response of the RCF samples, a metric was developed based on the relative standard deviation (RSD) in the Raman intensity response of the RCF samples. For both the 100 × 100 µm^2^ and 20 × 20 µm^2^ ROIs, two‐dimensional maps of the Raman intensity of individual pixels with respect to the nominal (mean) intensity over the ROI were created. The RSD in the Raman intensity response of the RCF samples was assessed over the 100 × 100 µm^2^ ROIs for each dose.

## 3 RESULTS

### 3.1 Raman spectra of RCFs

**FIGURE 2:**
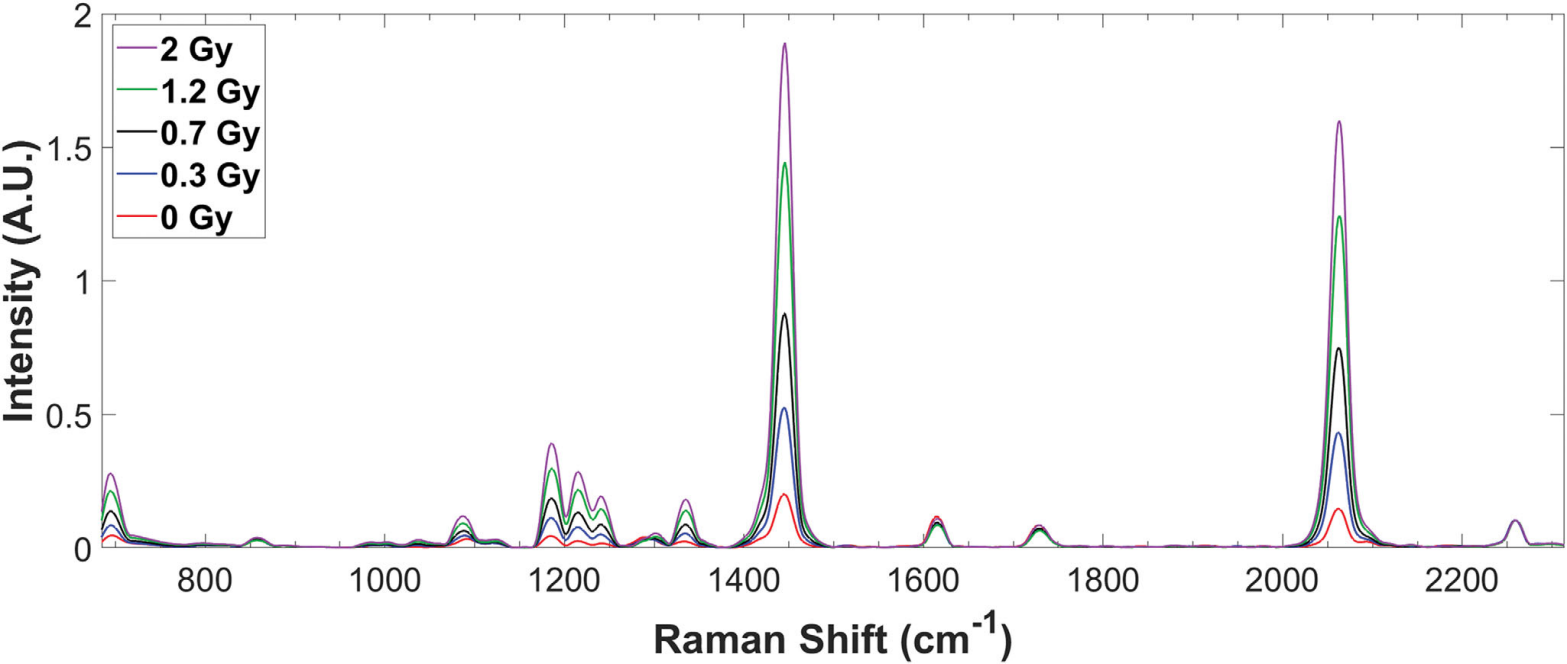
The mean (*n* = 500) of the Raman spectral response of RCFs measured with a 60× WI objective over the 100 × 100 µm^2^ scan area. Spectra after data preprocessing are shown for four representative doses and a control sample. WI, water immersion.

Figure 2a illustrates the mean of 500 preprocessed Raman spectra measured with a 60× WI objective of the custom Raman microscope over the 100 × 100 µm^2^ scan area in a 10 × 10 grid for representative dose values of 0.3, 0.7, 1.2, and 2 Gy. These correspond to a partially polymerized state due to the presence of monomer and polymer signals. The prominent peaks in the Raman spectrum at 696, 1086, 1445, and 2060 cm^−1^ are assigned respectively to vibrations of the δ(CCC), ν(C − C), ν(C = C) and ν(C ≡ C) modes in the backbone of the polyPCDA active layer. The peaks at 1186, 1216, 1240, and 1333 cm^−1^ are due to the CH_2_ wagging and twisting modes of the all‐*trans* alkyl chains.^1^ The peaks at 1614 and 1725 cm^−1^ are due to the stretching vibrations of the C = C and C = O bonds in the polyester laminate layer of the RCF.^26^ The peak at 2260 cm^−1^ is assigned to the vibration of the ν(C ≡ C) mode in the monomer crystals.^1^ Figure S1 shows that the Raman intensity of the 2260 cm^−1^ monomer peak does not vary significantly with dose. For example, even at 30 Gy, its value is within 0.46% of the mean Raman intensity for all doses. This demonstrates that the peak is not sensitive to ionizing radiation within the dose range investigated (0.2–2 Gy).

### 3.2 Dose response using the 2260 cm^−1^ Raman peak intensity for normalization

**FIGURE 3:**
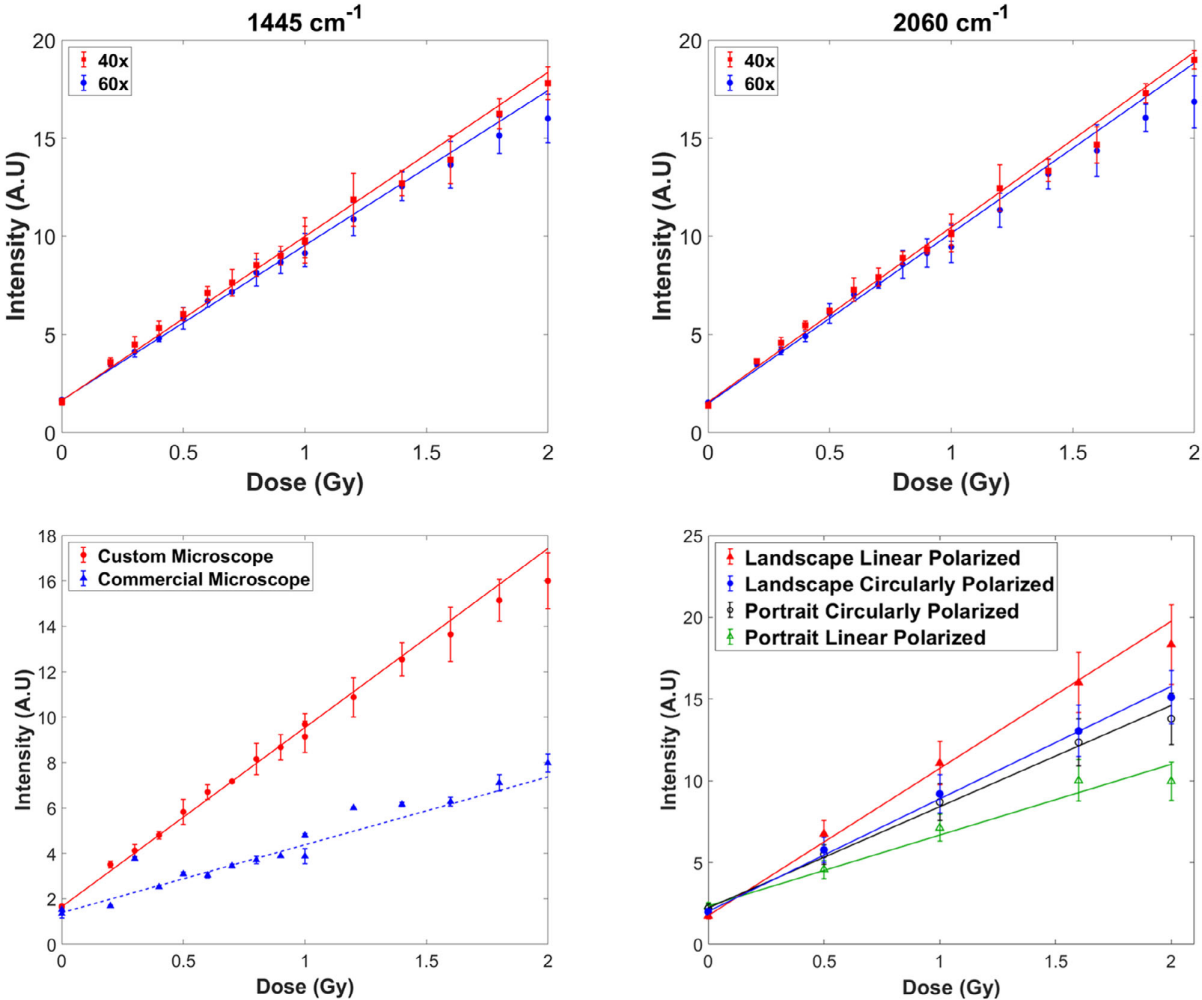
Dose response curves were generated using experimental data corresponding to the mean intensity values of the triplicate set of measurements for two different Raman peaks at (a) 1445 cm^−1^ and (b) 2060 cm^−1^ for 60× and 40× WI objectives of the custom Raman microscope. (c) Comparison of the average of three sets of data measured using the custom Raman microscope (60× WI objective) utilizing circularly polarized light versus the commercial Raman microscope (100× air objective) employing linearly polarized light. (d) Comparison of dose response curves for a representative set of doses at landscape and portrait orientations of the RCF samples using linearly polarized versus circularly polarized light. RCF, radiochromic films; WI, water immersion.

After normalizing the Raman spectra by the intensity of the monomer Raman peak at 2260 cm^−1^, the mean and standard deviation of the Raman spectral intensity were determined from the intensities at each pixel over the ROI. The Raman response over the triplicate sets of measurements was averaged and is illustrated in Figure 3a,b for the data taken with the 60× and 40× WI microscope objectives at the Raman shifts of 1445 and 2060 cm^−1^, respectively, which correspond to the highest intensity Raman active modes in the spectrum. The linear model proved adequate for our narrow 0.2–2 Gy dose range, offering computational and interpretive ease, unlike Mirza et al.’s broader 0–50 Gy range, where a nonlinear (exponential) model was applied.^17^, ^18^, ^19^ An average RSD of 5.5% and 5.0% was observed between the three sets for the 1445 cm^−1^ and 2060 cm^−1^ peaks, respectively. The standard deviation between identical samples within the datasets in triplicate was found to be 5.4%, 7.5% and 3.5% for the 60× WI custom microscope, 40× WI custom microscope, and commercial microscope, respectively. Three datasets consisting of spectra for the full range of doses were acquired daily over a 3‐day period for the 60× and 40× custom microscope setups. By contrast, for the commercial microscope, each of the three datasets for a dose was collected sequentially, with the total collection obtained over a 5‐day period. Hence, the lower uncertainty observed between datasets for each dose measured with the commercial microscope is likely explained by the subsequent similarity in measurement conditions, since the three measurements for each dose were collected within the span of 15 min.

Measurements of the films performed with a 40× WI objective yielded similar results to those with the 60× WI objective, showing a similar linear response to dose (Figure 3a,b). A two‐tailed *t*‐test was applied to each sample to test the similarity between the 40× and 60× data, which indicated, as expected, that the Raman response measured for each dose is consistent at a 95% confidence level for both 1445 and 2060 cm^−1^ peaks in the Raman spectrum.

The reproducibility of the Raman data was tested by acquiring additional measurements using the commercial Raman microscope fitted with a 100× air microscope objective. A comparison of the dose response curve to the data measured using the custom Raman microscope (60× water‐immersion microscope objective) is displayed in Figure 3c, which shows a distinct difference in slope between the two datasets. After further investigation, this was determined to be a result of differences in laser polarization between the custom and commercial Raman microscopes. A quarter waveplate installed in the custom microscope, circularly polarized the excitation laser light and thereby removed the dependence of the Raman intensity on the laser polarization, while the commercial microscope had a built‐in linearly polarized laser. Figure 3d shows the Raman intensity response for a representative set of irradiated samples (0, 0.5, 1.0, 1.6, 2.0 Gy) measured with linearly and circularly polarized light at portrait and landscape orientations. It is evident in Figure 3d that there is a significant difference in Raman response between both orientations for the linearly polarized light (e.g., an average difference of 45.6% at 2 Gy). In contrast, the mean intensities using circularly polarized light are within error bars (1 standard deviation) of the linear fit for each orientation. The small residual intensity difference is attributed to the slightly elliptical laser polarization, which could be further eliminated by ensuring perfect circular polarization of the laser. As such, utilizing circularly polarized light instead of linearly polarized light significantly reduced the difference in intensity between each orientation, thus minimizing the dependence of the Raman response of the films on orientation. Hence, the difference in slope between the data in Figure 3c is most likely due to the difference in polarization between the two Raman microscopes. It is consistent with the polarization‐dependent Raman intensity response of the EBT3 film reported earlier.^17^, ^18^ After testing with a separate set of EBT3 film samples, the Raman response of the film measured using the commercial microscope (Figure S2) was found to be sensitive to the orientation of the film, confirming this hypothesis. As seen in Figure 3c, the 0.3 Gy sample measurement with the commercial microscope yielded a Raman response more similar to the trend from the custom microscope. This suggests that the 0.3 Gy sample was inadvertently measured at a different orientation than the rest of the films. This reinforces the importance of utilizing circularly polarizing light to eliminate the orientation dependence of the films and minimize such measurement errors.

### 3.3 Dose homogeneity assessment based on two‐dimensional Raman intensity maps

**FIGURE 4:**
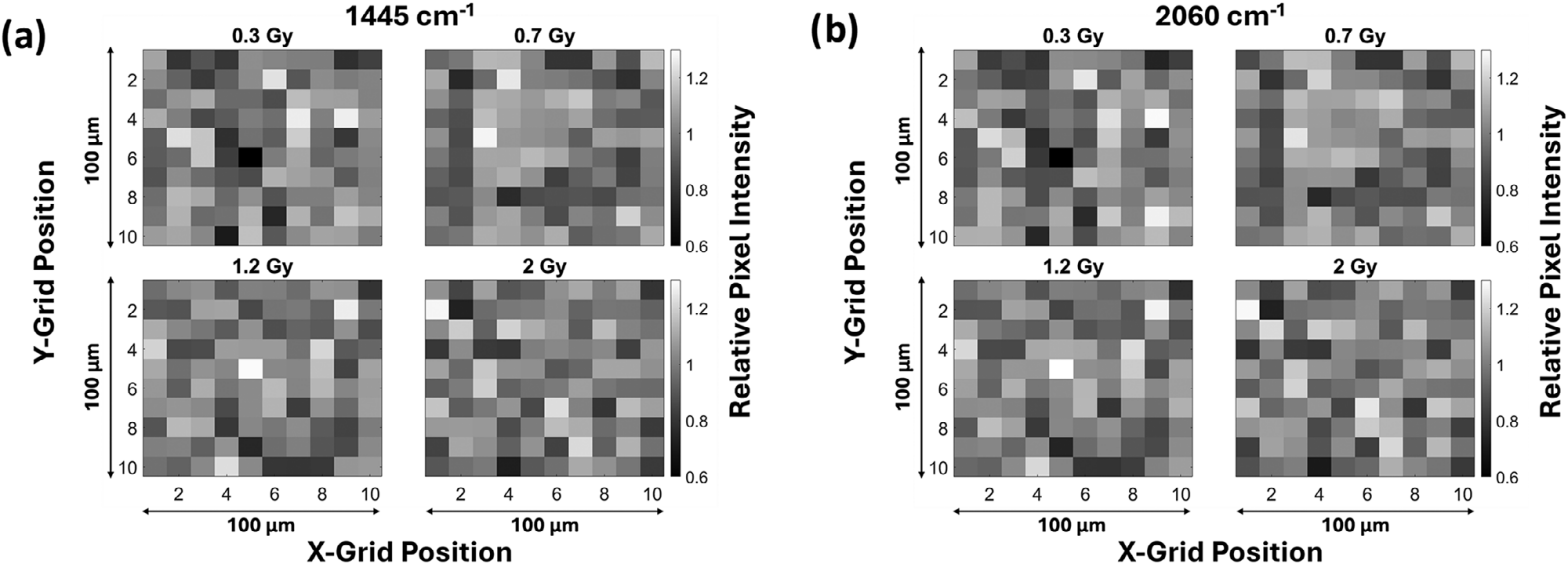
Two‐dimensional maps of the intensity of the Raman peaks at 1445 and cm^−1,^ normalized to the monomer peak at 2260 cm^−1^ consisting of 10 × 10 grids over 100 × 100 µm^2^ ROIs for a representative set of doses measured using a 60× WI objective on the custom microscope. ROI, region of interest; WI, water immersion.

The homogeneity of the Raman response was investigated by plotting the Raman intensity normalized to the 2260 cm^−1^ monomer peak across the 10 × 10 grid scans spanning a 100 × 100 µm^2^ area of the ROI. These showed significant variation in the relative Raman intensity of individual pixels compared to the nominal intensity of the ROI (Figure 4). RSD over the ROI was estimated based on the variation of the intensities of the pixels. The RSD values across the ROI are shown in Table 1 for all three focal volumes for a representative set of data for the intensity of the 1445 cm^−1^ Raman band. Table 1 shows that the RSD does not appear to be dependent on dose for any of the microscope setups (Tables S1, S2). It is also evident from Figure 4a,b that this dose‐independent heterogeneity in the two‐dimensional Raman intensity maps appears consistent for the 1445 and 2060 cm^−1^ Raman bands.

**TABLE 1:** Normalized intensity measurements of the 1445 cm^−1^ peak for a representative set of data from each microscope.

	Custom microscope (60×)			Custom microscope (40×)			Commercial microscope		
Dose (Gy)	Int^a^ (A.U)	SD (A.U)	RSD (%)	Int^a^ (A.U)	SD (A.U)	RSD (%)	Int^a^ (A.U)	SD (A.U)	RSD (%)
0	1.65	0.18	11.2	1.44	0.14	9.6	1.52	0.13	8.8
0	1.66	0.18	11.1	1.39	0.13	9.6	1.20	0.13	11.2
0.2	3.50	0.39	11.1	3.41	0.32	9.5	1.72	0.17	10.1
0.3	4.33	0.53	12.3	4.25	0.47	11.0	3.85	0.22	5.7
0.4	4.65	0.46	10.0	5.14	0.60	11.7	2.54	0.12	4.9
0.5	6.40	0.68	10.6	6.00	0.64	10.7	3.18	0.22	7.0
0.6	7.04	0.81	11.5	7.34	0.75	10.3	2.90	0.19	6.5
0.7	7.21	0.78	10.9	7.24	0.68	9.4	3.44	0.32	9.3
0.8	8.5	1.0	11.9	8.2	0.8	9.2	3.6	0.3	8.7
0.9	9.2	1.1	12.5	8.71	0.71	8.1	3.9	0.3	8.3
1.0	9.8	1.1	11.5	9.0	1.0	10.8	4.7	0.2	4.9
1.0	8.8	1.1	12.5	9.2	1.2	12.9	3.6	0.2	6.8
1.2	11.9	1.3	10.8	11.2	1.0	9.3	6.1	0.5	8.5
1.4	12.1	1.5	12.0	13.1	1.2	9.3	6.1	0.3	5.1
1.6	12.9	1.8	14.1	13.9	1.7	12.2	6.1	0.5	7.7
1.8	16.0	1.7	10.7	15.6	1.5	9.6	7.5	0.5	6.1
2.0	15.5	1.8	11.4	17.1	1.5	8.8	8.3	0.5	5.5

Abbreviations: RSD, relative standard deviation; SD, standard deviation of intensity over region of interest.

^a^Mean Raman intensity over the region of interest.

The average RSD across the 100 × 100 µm^2^ ROI is slightly less for the 40× WI objective (RSD ∼10.9%) compared to the 60× WI objective (RSD ∼11.3%) using the custom Raman microscope, and slightly lower for the commercial microscope (RSD ∼8.5%). This concurs with our expectation that the variation over the ROI increases as the size of individual pixels sampled by a microscope decreases.

**FIGURE 5:**
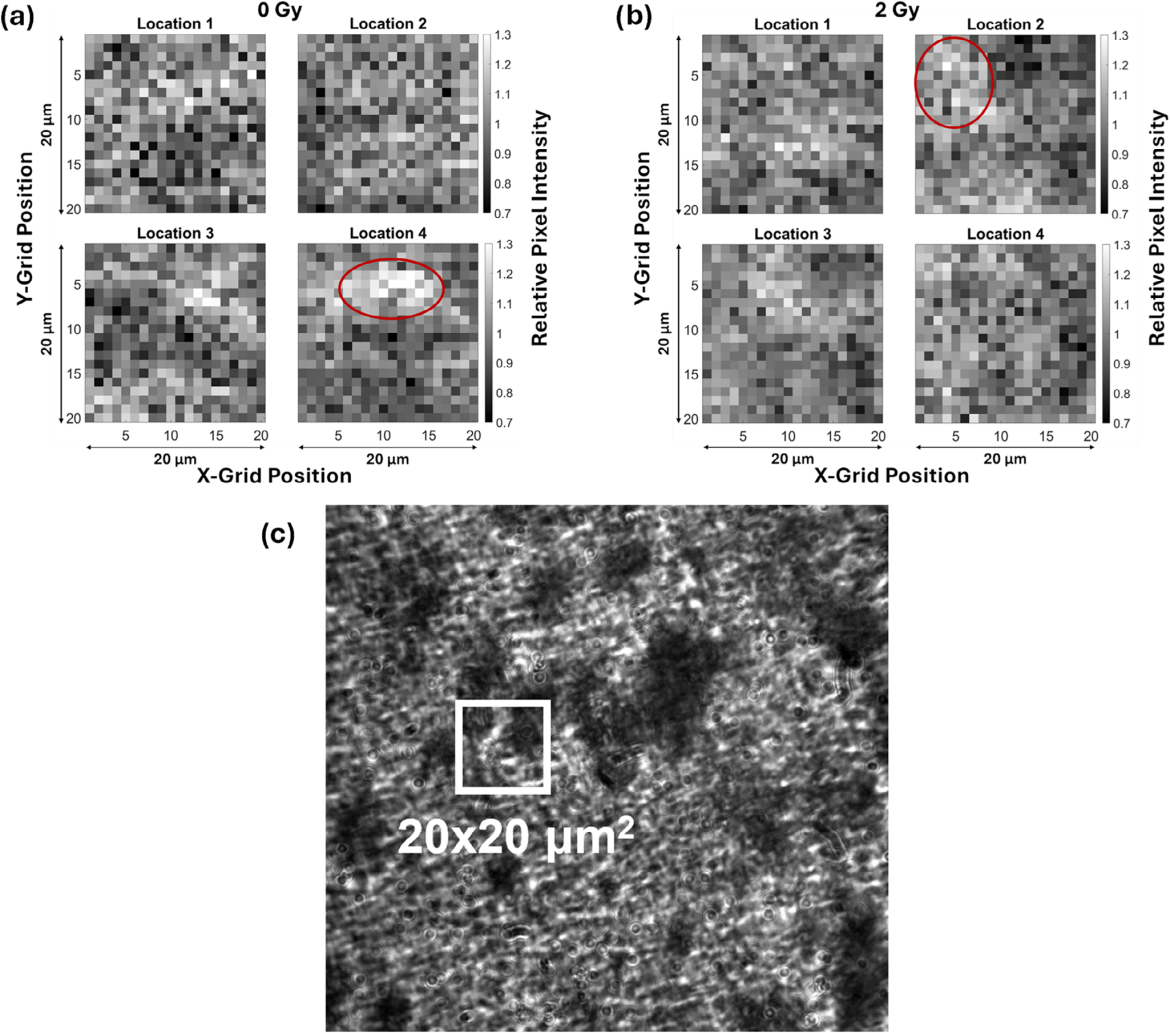
2D Raman intensity maps measured with the 60× WI objective on the custom microscope corresponding to the response of the 1445 cm^−1^ peak of 20 × 20 grids of control (0 Gy) (a) and 2 Gy films (b) for a 20 × 20 µm^2^ ROI. Red circles indicate areas corresponding to higher concentrations of polymer. (c) Brightfield transmission image of control film taken with the custom Raman microscope using a 40× WI objective with a representative 20 × 20 µm^2^ ROI for context. ROI, region of interest; WI, water immersion.

Each pixel intensity shown in Figure 4 corresponds to the value of the Raman intensity across a 1 µm‐wide pixel, but measured every 11 µm in the ROI of 100 × 100 µm^2^; it thus represents a coarse (∼11 µm) resolution Raman intensity map. To further investigate the source of the spatial heterogeneity in the Raman response, higher resolution grid scans of 20 × 20 µm^2^ ROIs were acquired as illustrated in the inset of Figure 1b, where the ∼1 µm‐wide pixels of the 20 × 20 µm^2^ scans are directly adjacent to one another. Figure 5 shows the corresponding results where the spatial heterogeneity in the form of bright and dark patches is observable in ∼5–10 µm^2^ sections in either image, irrespective of the dose value of 0 and 2 Gy. The length scale of these heterogeneous sections is consistent with the observed size of monomer crystals (mean width of 1.62 ± 0.35 µm and mean length of 9.4 ± 5.6 µm) previously reported in the literature.^27^ Moreover, the brightfield transmission image (Figure 5c) taken with the 40× WI objective using the custom Raman microscope also shows similar spatial heterogeneity that appears as patches of bright and dark regions in the image corresponding to minimum and maximum light absorption, respectively, in the active layer of the RCF samples. Such patches of bright and dark areas are not visible within the 100 × 100 µm^2^ ROIs (Figure 4) due to the lower (11 µm) resolution of these scans compared to the higher (1 µm) resolution of the 20 × 20 µm^2^ ROIs. Nevertheless, our results suggest that the underlying spatial heterogeneity in the PCDA monomer crystals in the active layer is the source of the spatially heterogenous Raman intensity response irrespective of the dose of exposure (Figure 5a,b), and explains our findings of the dose‐independent RSD value of ∼ 11% obtained from the 100 × 100 µm^2^ ROIs.

## 4 DISCUSSION

Here, we explored the potential of a method based on Raman micro‐spectroscopy of RCFs to generate reproducible datasets that are standardized for intra‐ and inter‐instrument comparisons. While Raman micro‐spectroscopy^20^ and confocal Raman spectroscopy^17^, ^18^, ^19^ have been applied to investigate RCFs in the past, in contrast to previous work, our current study was focused on developing a robust measurement technique for experimental microdosimetry which requires high sensitivity to low doses, high reproducibility and the ability to evaluate the spatial heterogeneity of the RCF response expected at low doses. The response of RCFs exposed to a low‐moderate dose range of x‐ray doses (0.2–2 Gy) was read out using a point‐scanning technique with high (e.g., 1 µm lateral and 6 µm axial) resolution. We used two different instruments with differing specifications for spatial resolution and laser polarization to determine the reproducibility in the response of RCFs. We found that increasing exposure to x‐ray dose results in a higher Raman signal (Figure 2) in the EBT3 RCF samples, as expected. This is because the quantity of radiation‐sensitive Raman active bonds in the polymer backbone increases with increasing radiation‐induced film polymerization. We used the Raman intensity of the monomer peak at 2260 cm^−1^ in the active layer (Figure 2) to normalize the Raman spectral intensity due to its insensitivity to dose over the 0–2 Gy dose range studied. For example, implementing this new normalization standard for the 60× data in Table 1 resulted in an r‐squared value of ∼0.98. This is better than the r‐squared value of ∼ 0.88 achieved after applying our previous technique that normalized the Raman spectrum to the Raman intensity of the 1626 cm^−1^ peak from the polyester laminate external to the active layer.^20^ Thus, normalization to an internal standard such as the monomer Raman peak at 2260 cm^−1^ within the active layer of the film reduces the influence of confounding factors during the measurement process, such as variations in focus over the ROI and thickness of the active layer. As demonstrated in this work, normalization to this internal standard also allows for comparisons between microscopes with different focal volumes that would result in differences in the contribution of the polyester to the active layer signal, thus affecting the normalization. To utilize this internal standard as a normalization technique, the 2260 cm^−1^ peak in the Raman spectrum must be detected by ensuring that data is collected to include Raman shifts up to 2300 cm^−1^.

The linearity of the dose response and sensitivity to dose are both desirable traits in experimental dosimetry. It is clear that the response curves determined from the data measured with the custom and commercial Raman microscopes are linear within the 0–2 Gy dose range for both the 1445 and 2060 cm^−1^ peaks (Figure 3a,b). The nonlinear exponential trend of the data fit observed in other studies, which examined a similar dose range^17^, ^18^, ^19^ was absent in our results. The linear dose‐response trend observed within the 0–2 Gy range can be attributed to the dose‐insensitive internal standard used for normalization of the active layer spectrum. On average, the results from measurements taken with our 40× and 60× WI objectives are consistent with one another at a 95% confidence level (Figure 3a,b). This suggests the dose response was consistent after normalization despite differences in the focal volume between the 40× and 60× WI objectives.

The Raman micro‐spectroscopy technique demonstrated here has the potential for reading out the dose‐response of RCFs even below 0.2 Gy, irrespective of the Raman instrument utilized for measurements, provided that careful attention is paid to the polarization of the incident light. We found that differences in laser polarization between Raman microscopes can be a significant confounding factor when comparing sets of measurements. For example, a difference in slope (∼2×) attributed to the difference in laser polarization was observed in Figure 3c between the dose response curves obtained using the custom (60× WI) and commercial (100× air) Raman microscopes. This is consistent with results reported by Mirza et al.,^17^, ^18^ where differences in the intensity of the Raman response were measured when RCF samples were mounted in the landscape versus portrait orientation. Minimizing the orientation dependence using circularly polarized light in the custom Raman microscope (Figure 3d) increased the reproducibility of the Raman measurements between microscope setups. Although a randomly polarized multimode laser could also be used for polarization‐insensitive Raman measurements, such as reported in our previous work,^20^ the setup will not provide ∼1 µm spatial resolution, limiting applications in experimental microdosimetry.

We found a significant spatial heterogeneity in the film Raman intensity response over the 100 × 100 µm^2^ ROI (Figure 4a,b). The relative intensity of individual pixels within the 100 × 100 µm^2^ ROI varied significantly, from as low as ∼60% to as high as 130% with respect to the nominal intensity over the ROI. This heterogeneity in the Raman signal is still significant despite implementing normalization to the internal standard at 2260 cm^−1^ and vector normalization. RSD of the Raman signal over the ROI was ∼ 11%, independent of the dose of exposure in the 0–2 Gy range for the 60× WI objective (Figure 4 and Table 1). From previous work involving Monte Carlo simulations, the variation in energy deposition (due to the stochastic nature of radiation transport and energy deposition) in the RCFs is expected to be ∼ 91% at 0.2 Gy,^25^ and reduces to 27% at 2 Gy for a 1 µm side length cubic voxel. Our results did not show the variation in the response of the RCFs that would correspond to variations in specific energy distribution in (1 µm^3^) volumes expected from such Monte Carlo simulations of the heterogeneous energy deposition. Further investigation of the higher resolution 2D Raman intensity maps of the 20 × 20 µm^2^ ROI (Figure 5) revealed regions of heterogeneity on a similar length scale to the known size of the monomer crystals (mean width of 1.62 ± 0.35 µm and mean length of 9.4 ± 5.6 µm) in both the 0 and 2 Gy irradiated RCF samples.^27^ This was analogous to the areas of inhomogeneity seen in the brightfield transmission image (Figure 5c) of an unirradiated RCF sample.^1^

Taken together, our results suggest that the spatial heterogeneity in the Raman intensity distribution across a 100 × 100 µm^2^ ROI in the 0–2 Gy dose range is due to the heterogeneity in the density of the polymerized PCDA that in turn arises from the inhomogeneity in the dispersion of monomer crystals in the active layer of the RCF. This will result in significant uncertainty when estimating the specific energy (stochastic analogue of dose) at a micrometer scale, for example, to a singular biological cell at low‐moderate doses of radiation (< 0.1–2 Gy). This is a limiting factor for the utility of EBT3 films for experimental microdosimetry since significant variability in the spatial distribution of specific energy is expected due to the stochastic nature of energy deposition at low doses.^25^ Thus, the spatial heterogeneity of the energy deposition and film heterogeneity are both contributing to the total observed heterogeneity of the 2D Raman intensity map. Statistical approaches that can decouple the spatial heterogeneity of the films from the spatial heterogeneity of the energy deposition are warranted. Our ongoing work focuses on developing a statistical modelling framework to extract the small spatially heterogenous Raman intensity response at low doses induced by the ionizing radiation from the comparatively large background. The background is the result of initial polymerization of the films (i.e., 0 Gy signal) and includes the inherent spatial heterogeneity in the distribution of polymerized PCDA in the RCF. The estimated spatial heterogeneity of the energy deposition from the Raman intensity maps from our experimental datasets will then be compared to the results from Monte Carlo simulations of the microdosimetric spread at the same doses for validation. Alternatively, the Raman micro‐spectroscopy read‐out technique demonstrated here could be applied to alternative 2D materials that are more spatially homogenous compared to RCFs.

## 5 CONCLUSIONS

This work explored the feasibility of a Raman micro‐spectroscopy‐based read‐out technique utilizing EBT3 RCFs. This is a step toward generating data for experimental microdosimetry. The 2260 cm^−1^ monomer peak in the RCF active layer was utilized as an internal standard to maximize differences between doses within a dataset. The use of the internal standard resulted in a dose‐response curve that was linear and sensitive, representing an improvement over previous work that used the polyester laminate signal as a normalization standard instead. Measurements using different Raman micro‐spectroscopy setups indicated that polarization of the excitation source has a significant impact on the film response and should be minimized to properly compare different setups. Thus, the experimental setup should use a circularly polarized light source, and the measurement protocol should include Raman data collection up to 2300 cm^−1^ to detect the peak at 2260 cm^−1^. This work spatially mapped out the Raman response of the RCFs over a 100 × 100 µm^2^ ROI, illustrating the influence of the heterogeneity in the films on the read‐out at micrometer‐scale resolution. In addition, higher resolution maps of the intensity over a higher resolution 20 × 20 µm^2^ ROI were produced that illustrate that the variation in the Raman read‐out of these films is likely a result of heterogeneity in the distribution of PCDA crystals throughout the active layer in the films.

## CONFLICT OF INTEREST STATEMENT

The authors declare no conflicts of interest.

## Supporting information



Supplementary Information
