# Supplementary material for: Exploring the potential of Raman micro‐spectroscopy of radiochromic films for experimental microdosimetry
Source: Med Phys. 2025 Jul 15;52(7):e17900. doi: 10.1002/mp.17900 (PMC12264321; doi:10.1002/mp.17900)
Supplement: Supplementary file 1 — Supplementary Information [file MP-52-0-s001.docx]

Table S1: Normalized intensity measurements of the 1445 cm^-1^ peak for second set of data from each microscope

|  |  | Custom Microscope (60X) | | |  | Custom Microscope (40X) | | |  | Commercial Microscope | | |
| --- | --- | --- | --- | --- | --- | --- | --- | --- | --- | --- | --- | --- |
| Dose (Gy) |  | Int^a^ (A.U) | SD (A.U) | RSD (%) |  | Int^a^ (A.U) | SD (A.U) | RSD (%) |  | Int^a^ (A.U) | SD (A.U) | RSD (%) |
| 0 |  | 1.63 | 0.22 | 13.7 |  | 1.61 | 0.14 | 8.5 |  | 1.55 | 0.11 | 7.1 |
| 0 |  | 1.72 | 0.29 | 16.9 |  | 1.72 | 0.16 | 9.1 |  | 1.27 | 0.16 | 12.8 |
| 0.2 |  | 3.65 | 0.40 | 10.9 |  | 3.84 | 0.36 | 9.3 |  | 1.70 | 0.12 | 7.3 |
| 0.3 |  | 4.20 | 0.51 | 12.1 |  | 4.94 | 0.45 | 9.0 |  | 3.71 | 0.20 | 5.5 |
| 0.4 |  | 4.98 | 0.52 | 10.5 |  | 5.73 | 0.56 | 9.7 |  | 2.46 | 0.14 | 5.6 |
| 0.5 |  | 5.76 | 0.68 | 11.8 |  | 6.38 | 0.65 | 10.2 |  | 3.02 | 0.14 | 4.8 |
| 0.6 |  | 6.64 | 0.80 | 12.0 |  | 6.86 | 0.64 | 9.3 |  | 3.12 | 0.16 | 5.2 |
| 0.7 |  | 7.09 | 0.80 | 11.3 |  | 8.42 | 0.82 | 9.8 |  | 3.36 | 0.22 | 6.5 |
| 0.8 |  | 8.6 | 1.0 | 11.9 |  | 9.2 | 0.9 | 9.8 |  | 3.7 | 0.2 | 5.9 |
| 0.9 |  | 8.0 | 0.9 | 11.4 |  | 9.56 | 1.13 | 11.8 |  | 3.9 | 0.3 | 9.0 |
| 1 |  | 10.1 | 1.2 | 11.9 |  | 11.1 | 1.1 | 10.2 |  | 4.8 | 0.3 | 6.2 |
| 1 |  | 9.9 | 1.0 | 10.3 |  | 10.6 | 1.0 | 9.0 |  | 3.8 | 0.2 | 6.4 |
| 1.2 |  | 10.5 | 1.2 | 11.0 |  | 13.4 | 1.2 | 8.7 |  | 6.0 | 0.3 | 5.5 |
| 1.4 |  | 13.4 | 1.6 | 12.3 |  | 13.0 | 1.3 | 10.3 |  | 6.2 | 0.4 | 6.2 |
| 1.6 |  | 15.0 | 1.6 | 10.4 |  | 15.1 | 1.4 | 9.5 |  | 6.4 | 0.4 | 6.1 |
| 1.8 |  | 15.3 | 1.9 | 12.4 |  | 17.1 | 1.8 | 10.5 |  | 7.1 | 0.4 | 6.0 |
| 2 |  | 17.4 | 2.1 | 11.8 |  | 18.7 | 1.7 | 9.2 |  | 8.1 | 0.5 | 6.2 |

^a^Mean Raman intensity over region of interest

Table S2: Normalized intensity measurements of the 1445 cm^-1^ peak for third set of data from each microscope

|  |  | Custom Microscope (60X) | | |  | Custom Microscope (40X) | | |  | Commercial Microscope | | |
| --- | --- | --- | --- | --- | --- | --- | --- | --- | --- | --- | --- | --- |
| Dose (Gy) |  | Int^a^ (A.U) | SD (A.U) | RSD (%) |  | Int^a^ (A.U) | SD (A.U) | RSD (%) |  | Int^a^ (A.U) | SD (A.U) | RSD (%) |
| 0 |  | 1.59 | 0.16 | 9.8 |  | 1.58 | 0.15 | 9.2 |  | 1.49 | 0.13 | 8.4 |
| 0 |  | 1.63 | 0.18 | 11.2 |  | 1.58 | 0.24 | 15.3 |  | 1.56 | 0.10 | 6.1 |
| 0.2 |  | 3.36 | 0.35 | 10.5 |  | 3.52 | 0.36 | 10.2 |  | 1.63 | 0.10 | 6.3 |
| 0.3 |  | 3.82 | 0.52 | 13.6 |  | 4.26 | 0.38 | 8.9 |  | 3.75 | 0.25 | 6.6 |
| 0.4 |  | 4.77 | 0.47 | 9.8 |  | 5.11 | 0.49 | 9.5 |  | 2.55 | 0.16 | 6.4 |
| 0.5 |  | 5.31 | 0.68 | 12.9 |  | 5.72 | 0.59 | 10.3 |  | 3.06 | 0.16 | 5.4 |
| 0.6 |  | 6.42 | 0.76 | 11.9 |  | N/A | N/A | N/A |  | 3.08 | 0.27 | 8.9 |
| 0.7 |  | 7.21 | 0.87 | 12.0 |  | 7.23 | 0.71 | 9.8 |  | 3.52 | 0.18 | 5.1 |
| 0.8 |  | 7.4 | 1.0 | 13.5 |  | 8.22 | 0.63 | 7.6 |  | 3.9 | 0.2 | 5.9 |
| 0.9 |  | 8.8 | 1.1 | 12.0 |  | 8.8 | 0.9 | 10.7 |  | 3.9 | 0.2 | 5.8 |
| 1 |  | 9.1 | 1.0 | 11.2 |  | 9.27 | 0.90 | 9.7 |  | 4.8 | 0.3 | 6.1 |
| 1 |  | 8.6 | 1.1 | 13.1 |  | 9.3 | 0.8 | 9.1 |  | 4.2 | 0.2 | 5.1 |
| 1.2 |  | 10.2 | 1.2 | 11.7 |  | 11.0 | 1.2 | 10.8 |  | 6.0 | 0.4 | 7.4 |
| 1.4 |  | 12.1 | 1.4 | 11.8 |  | 12.0 | 1.2 | 10.3 |  | 6.1 | 0.4 | 7.1 |
| 1.6 |  | 13.0 | 1.5 | 11.9 |  | 12.6 | 1.4 | 11.0 |  | 6.3 | 0.4 | 6.5 |
| 1.8 |  | 14.1 | 1.6 | 11.5 |  | 16.0 | 1.8 | 11.3 |  | 6.8 | 0.4 | 5.9 |
| 2 |  | 15.1 | 2.0 | 13.1 |  | 17.6 | 1.8 | 10.1 |  | 7.5 | 0.4 | 4.9 |

^a^Mean Raman intensity over region of interest


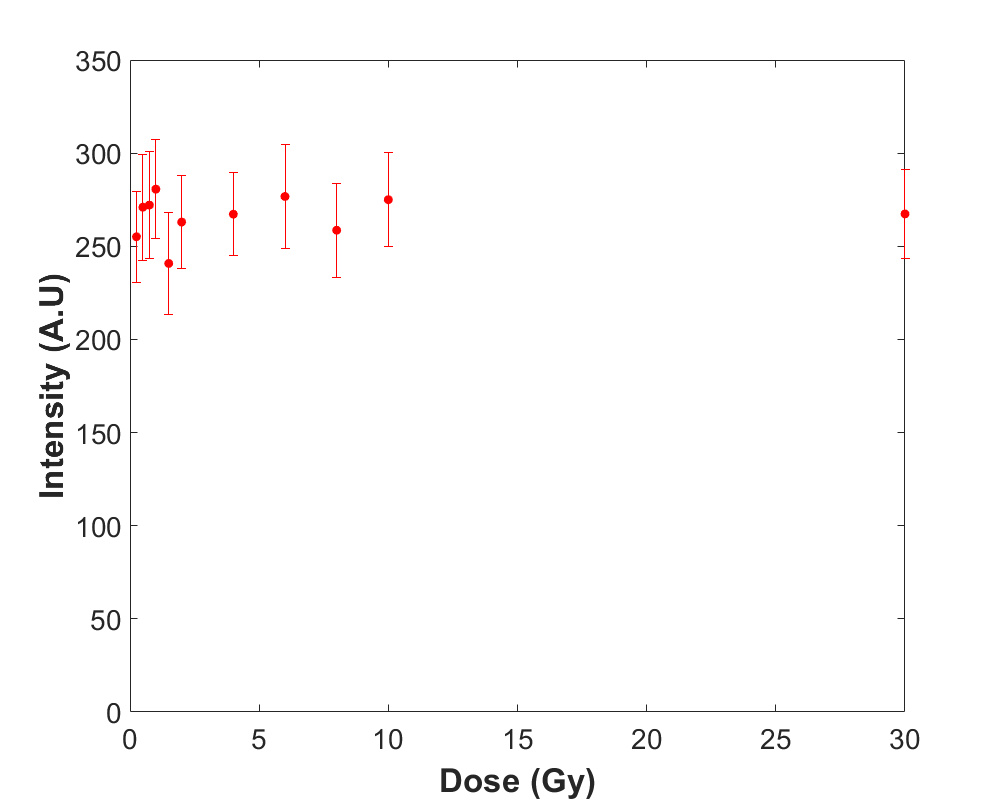


Fig S1**:** Raman intensity of the peak at 2260 cm^-1^ attributed to monomer crystals in the RCF active layer measured for a representative set of doses (0.25, 0.5, 0.75, 1, 1.5, 2, 4, 6, 8, 10, 30 Gy).


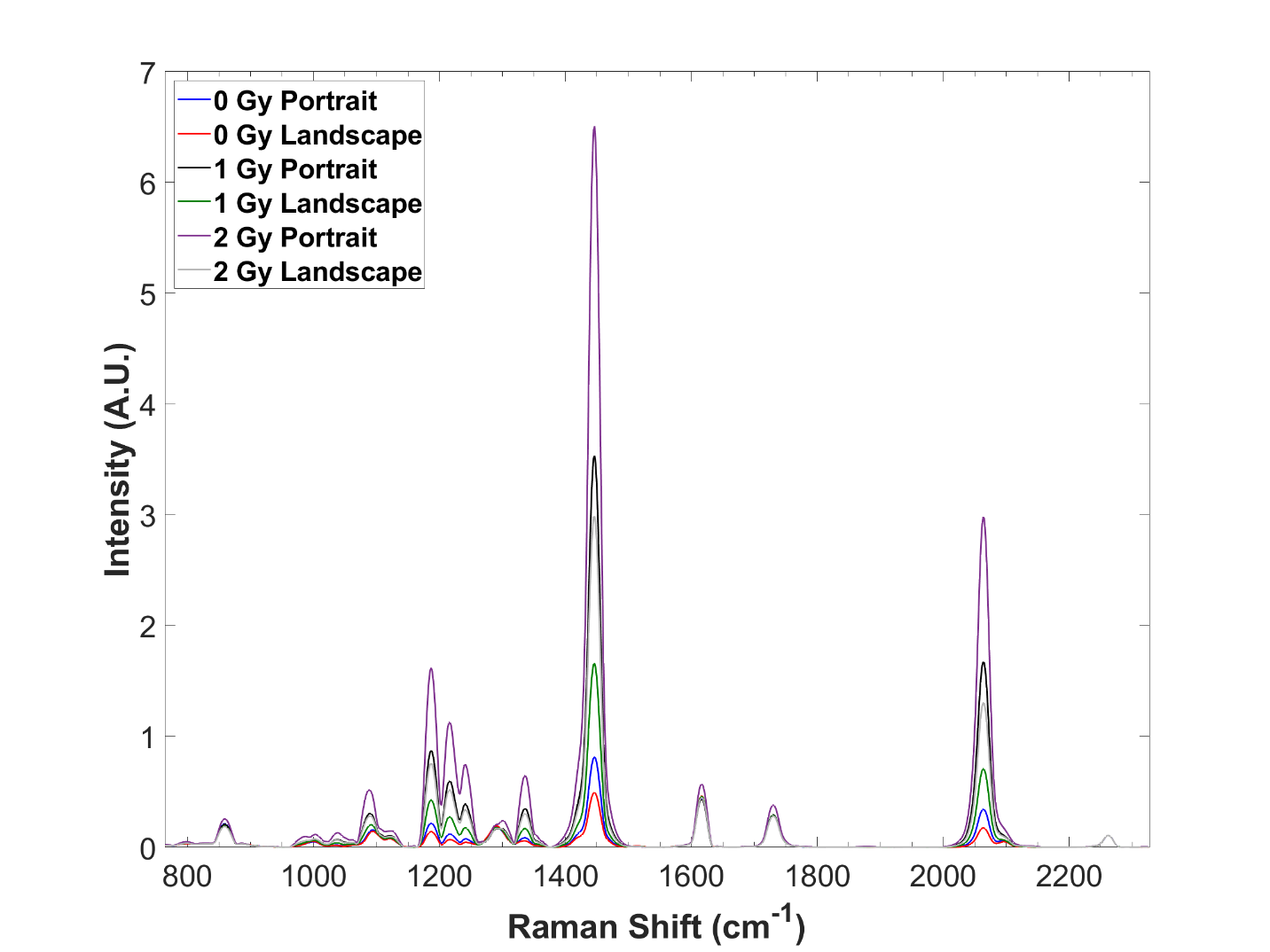


Fig S2: The mean (n=100) Raman spectral response of EBT3 films measured using commercial microscope at portrait versus landscape orientations. Spectra after data preprocessing are shown for three representative doses (0, 1 and 2 Gy).
